# The Story of the Insane from Year to Year

**Published:** 1898-11-12

**Authors:** 


### THE STORY OF THE INSANE FROM YEAR TO YEAR.

(Eleventh Series.)

### DORSET COUNTY ASYLUM.

In this asylum there was an increase of only six patients during the year. The admissions were 125, the readmissions 17, the recoveries 49, and the deaths 53. Calculated on the admissions, and excluding patients transferred from other asylums, the recoveries stand at 42·24 per cent.; and, calculated on the average number resident, the deaths stand at 7·49 per cent. Sixteen of the deaths were caused by cerebral and spinal diseases, 6 by consumption, 2 by pneumonia, and 1 by peritonitis. Table X. shows the causes of insanity in those admitted, and we learn from it that 18 of the patients were driven insane by mental worry, domestic troubles, and adverse circumstances, 11 by intemperance in drink, and 40 by hereditary influence. Dr. Macdonald reviews once again the well-ventilated question of the real or supposed increase of insanity at the present day, and he arrives at the unpopular, but, we believe, the true opinion, that there is an increase. The accumulation theory is a very nice one, and it will account for much, but he much doubts if it will account for all. When speaking of the causation of insanity, Dr. Macdonald states that his own observations lead him to believe that 50 per cent. of the insane in the County of Dorset were foredoomed by heredity, and he adds that if unsuitable marriages could be prevented the tendency would be avoided to a large extent. It is well known that the training of attendants and nurses is carefully carried out in this asylum, and we believe that the only correct system of examination is followed, namely, that by the medical officers of the asylum, and that a three years' course of training is required. If Dr. Macdonald adhere to this system, and keep up or advance the standard of efficiency, he may feel quite sure that his efforts will be appreciated, and his nurses placed on at least as high a level as the nurses of ordinary hospitals. The visitors say that the medical superintendent has given "the amplest grounds for confidence in his management of the asylum, and in his care and treatment of the patients." There are about 80 private patients here, and these pay from 10s. to 42s, a week each. The average cost per head per week of the county patients is 9s. 0½d.

### MULLINGAR DISTRICT ASYLUM.

Here the limits of accommodation are stated to be only 621, but 712 were in the asylum on the first day of 1897, and this number had increased to 742 by the end of the year, so that the asylum must have begun its work for 1898 with 121 patients more than it was intended for. There were 121 admissions, 39 readmissions, 40 recoveries, 15 discharged relieved, and 72 deaths. The percentage of recoveries, calculated on the admissions, stands at 25, and that of the deaths 9·9 on the average numbers resident. We notice that 20 of the deaths were caused by consumption and 26 by pneumonia, while cerebral diseases account for 11 only. The pneumonia was of septic nature, and proved fatal to about half of those attacked. Of the admissions 15 owed their insanity to various moral causes. Drink accounts for 9, and hereditary influence to 35. The overcrowding will be to some extent relieved by the opening of a temporary wooden structure, and it is proposed to build an annexe for 130 patients. Dr. Finnigan has a lady assistant medical officer permanently on the staff, and the office is at present held by Dr. Gertrude Grogan, a graduate of the Royal University. The matron, after sixteen years' service, resigned on a pension, and Dr. Finnigan has wisely divided the duties of this office and appointed a head nurse and a housekeeper.

